# 

**Published:** 1903-12

**Authors:** 


### CONTENTS.

ORIGINAL COMMUNICATIONS—

PATHOLOGY AND SYMPTOMS OF CONSTIPATION. By Mark W. Peyser, M.D., Richmond, Va.... 577
PUERPERAL INSANITY. By J. W. Palmer, M.D., Alley, Ga.... 581
PATHOLOGY AND TREATMENT OF SOME OF THE AFFECTIONS OF THE UPPER AIR TRACT. By James M. Crawford, M.D., Atlanta, Ga.... 590
SOME INDICATIONS FOR THE USE OF NORMAL SALINE SOLUTION. By J. B. Morgan, M.D., Augusta, Ga.... 596
AN OPERATION FOR PERMANENT AND QUICK CURE OF HEMORRHOIDS, ALMOST BLOODLESS AND PAINLESS. By John C. McAfee, A.B., M.D., Macon, Ga.... 601
THE TREATMENT OF GONORRHEA. By S. D. Warnock, M.D., Atlanta, Ga.... 605

EDITORIAL NOTES AND COMMENTS—

DEATH OF DR. JAMES MCFADDEN GASTON.... 608
A MEDICAL MEDITATION.... 611
SOUTHERN SURGICAL AND GYNECOLOGICAL ASSOCIATION MEETING.... 613
THE WEATHER COCK.... 616

CONTENTS CONTINUED.... X

